# Vector transmission and epidemiology of ‘*Candidatus* Phytoplasma pyri’ in Austria and identification of *Cacopsylla pyrisuga* as new pathogen vector

**DOI:** 10.1007/s41348-021-00526-y

**Published:** 2022-02-06

**Authors:** Monika Riedle-Bauer, Caroline Paleskić, Christina Schönhuber, Martina Staples, Günter Brader

**Affiliations:** 1 Federal College and Research Institute for Viticulture and Pomology Klosterneuburg, Wienerstraße 74, 3400 Klosterneuburg, Austria; 2 Austrian Institute of Technology, Konrad-Lorenz-Straße 24, 3430 Tulln, Austria

**Keywords:** Pear decline, Pear psyllid, Transmission experiment, *Cacopsylla pyri*, *C. pyrisuga*, *C. pyricola*, aceF, imp

## Abstract

Pear decline, induced by the phytoplasma '*Candidatus* Phytoplasma pyri', transmitted by pear psyllids, is one of the most devastating diseases on *Pyrus communis* in Europe and North America. Investigations of pear psyllids in 4 pear orchards in lower Austria showed the presence of *Cacopsylla pyri*, *C. pyricola* and *C. pyrisuga* at all locations. PCR analyses revealed overall phytoplasma infection rates for *C. pyri* of 5.4%, for *C. pyricola,* of 4.6%, for *C. pyrisuga* remigrants of 9.6% and for *C. pyrisuga* emigrants of 0%. The rates of PCR-positive *C. pyri* and *C. pyricola* individuals varied greatly in the course of the year, and the highest infection rates were observed in late summer, autumn and in late winter. In transmission experiments with healthy pear seedlings, winterform individuals of *C. pyri and C. pyricola* transmitted the pathogen to 19.2% (5 out of 26) and 4.8% (2 out of 41) of the test plants, respectively. The vectoring ability of *C. pyrisuga* was experimentally proven for the first time, and in transmission experiments with remigrants, 9.5% (2 out of 21) of the pear seedlings were infected. Our data indicate a significant risk of pathogen transmission in pear orchards during the greater part of the year, especially in late winter, early spring and autumn. Multilocus sequence analysis by aid of the genes *aceF* and *imp* allowed the discrimination between 15 phytoplasma types. Three so far undescribed *aceF* genotypes and four undescribed *imp* genotypes were identified.

**Supplementary Information:**

The online version contains supplementary material available at 10.1007/s41348-021-00526-y.

## Introduction

Pear decline (PD), induced by the phytoplasma '*Candidatus* Phytoplasma pyri', is one of the most devastating diseases on *Pyrus communis* in Europe and North America (Seemüller et al. 2011). Based on sequence analysis of 16S rDNA, ‘*Ca*. P. pyri’ belongs to the apple proliferation group 16SrX together with other important fruit tree phytoplasmas, such as ‘*Ca*. P. mali’ and ‘*Ca*. P. prunorum’ (Seemüller and Schneider 2004). Phytoplasmas from this ribosomal group cause severe disorders in pome and stone fruits worldwide.

Severe losses in pear production induced by '*Ca.* P. pyri’ were for the first time reported in Italy and North America in the 1940s and 1950s (Jensen et al. 1964; Bertaccini and Duduk 2013). Currently, the pathogen is present in nearly all pear growing regions throughout Europe, in North and South America as well as in parts of Northern Africa (CABI 2020).

'*Ca.* P. pyri' infects most or all species of the genus *Pyrus* and also quince (*Cydonia oblonga*). The latter is frequently used as rootstock in pears (Seemüller and Schneider 2004; EPPO 2019). PD develops in two different forms: quick decline leads to a sudden death of the entire tree within a few weeks, whereas slow decline is characterised by a continuous degradation and death after some years. Quick or slow disease progression is attributed to the susceptibility of the rootstocks (Seemüller et al. 1986, 1998; Giunchedi et al. 1995; Pastore et al. 1997). Slow decline generally becomes noticeable in late summer or autumn when affected trees show premature leaf reddening, leaf rolling and early leaf fall. Further disease symptoms may vary in severity. Generally, infected trees are characterised by fewer, smaller and leathery leaves, reduced terminal growth, diminished fertility and fruit size and eventually, death of trees. A necrotic phloem ring at the graft union may develop (EPPO 2019; Seemüller et al. 1984a, b). In Austria, pear decline is widespread (Steffek et al. 2011) and causes severe losses in intensive as well as extensive pear orchards. The main cultivar in intensive pear cultivation in Austria is cv. ‘Williams’ (around 40% of the total production, Statistics Austria 2019), a cultivar considered as very susceptible to pear decline (EPPO 2019). In extensive plantings, many traditional cultivars for the production of perry, e.g. 'Speckbirne', are grown, which are susceptible to ‘*Ca*. P. pyri’ infections (A. Ennser, personal communication).

Jumping plant lice (Genus *Cacopsylla*, Hemiptera, Psyllidae) are considered to be epidemiologically relevant insect vectors of '*Ca.* P. pyri' (Jarausch et al. 2019a). A large number of *Cacopsylla* species feeding on pear have been recorded in the Palearctic zone (Cho et al. 2017). Apart from weakening the trees by excessive sap sucking and spoiling the fruits by secreting honeydew (Cho et al. 2017), several species have been associated with transmission of ‘*Ca*. P. pyri’. Successful phytoplasma transmission experiments with *C. pyricola* (Foerster, 1848) as vectoring species have been carried out in North America (Jensen et al. 1964) and the UK (Davies et al. 1992). The vectoring ability of *C. pyri* (Linné, 1758) was confirmed by transmission tests in France, Italy and Spain (Lemoine 1991; Carraro et al. 1998; Garcia Chapa et al. 2005). In addition, two other psyllids, namely *C. pyrisuga* (Foerster, 1848) and *C. bidens* (Šulc, 1907) have been found to carry the phytoplasma, but successful transmission experiments have not been reported so far (Jarausch et al. 2019a). In Austria, three confirmed or putative phytoplasma vectors, namely *C. pyri, C. pyri*cola and *C. pyrisuga,* have been recorded (Lethmayer et al. 2011).

*C. pyri, C. pyricola* and *C. bidens* are polyvoltine and can be found on pear trees all year round. All three species are seasonally dimorphic, producing a large, dark overwintering form and a smaller, lighter summerform (Burckhardt and Hodkinson 1986). All three species predominantly overwinter as adults in bark crevices of the trees, in case of *C. pyricola;* however, the winterform has also been recorded outside pear orchards (Ossiannilsson 1992; Horton et al. 1994). The number of insect generations depends on the climatic zone; for *C. pyri* 2–8, for *C. pyricola* 3–5 generations per year have been reported (Hodkinson 2009; Garcia Chapa et al. 2005; Civolani 2012; Jarausch et al. 2019a). In contrast, *C. pyrisuga* is an univoltine migratory species. At the end of winter or in early spring, the adults migrate to *Pyrus* spp. where they lay eggs and the immature stages develop. The new generation adults quit their *Pyrus* developmental hosts and spend the rest of the year until the next spring on their overwintering shelter plants (conifer species) (Ossiannilsson 1992; Jarausch et al. 2019a).

One way of improving insight into phytoplasma epidemiology is a multilocus sequence analysis of the pathogen types involved in disease outbreaks. Comparison of phytoplasmas in host plants and insect vectors both on a global and on a local scale enables conclusions on pathogen dispersal, allows to trace the route of propagation of phytoplasma types and finally helps to develop well-adapted control strategies. A multilocus sequence analysis approach analysing the genetic diversity of the three temperate fruit tree phytoplasmas ‘*Ca.* P. prunorum’, ‘*Ca*. P. mali’ and ‘*Ca.* P. pyri’ based on the genes *aceF, pnp, secY* and *imp* has been established (Danet et al. 2011). Among the four genetic loci used in this and a Slovenian study (Pavšič et al. 2014), the genes *imp* encoding for an immunodominant surface protein and *aceF* encoding for the dihydrolipoamide acyltransferase, a protein involved in glycolysis, showed the highest variability and discriminative power, which is of particular interest for discrimination of local populations. A high diversity of the *imp* gene with 12 different genotypes in ‘*Ca.* P. pyri’ was recently described also in the Czech Republic (Bohunicka et al. 2018). In a recent Austrian study in apricots, molecular characterisation based on the genes *aceF* and *imp* discriminated between 10 ‘*Ca.* P. prunorum’ types and allowed to trace the propagation paths of the pathogen (Riedle-Bauer et al. 2019). Consequently, *imp* and *aceF* have been used here as markers to characterise Austrian ‘*Ca.* P. pyri’ strains.

The aim of the current study was to get new insights into epidemiology of Pear decline in Austria. The presence and the population dynamics of pear psyllids in pear orchards in Austria were determined, and their infection rates in the course of the year were analysed. The vectoring ability and the seasonal transmission efficiency of the present pear psyllid species were investigated in transmission experiments under controlled conditions. A finer characterisation of ‘*Ca*. P. pyri’ types present in plants and pear psyllids was achieved by sequence analysis of the two discriminative molecular markers *aceF* and *imp.*

## Materials and methods

### Test and sampling locations

Insect sampling for the investigation of population dynamics and for transmission experiments was carried out in 4 pear orchards in Lower Austria, namely Thallern, Rührsdorf 1, Rührsdorf 2 and Klosterneuburg (Table 1). Plant samples were collected from 8 different pear orchards in Eastern Austria as illustrated in Table 1.

**Table 1:** Location and description of sampling sites

Abbreviation	Location	Variety/Rootstock	Training system	Tree age (y)	Farming system	Identified phytoplasma types*
THA	Lower Austria; Thallern	Bosc/Fox11	Hedge, stem height 50–60 cm	4	Integrated	ATPYR1 ATPYR2 ATPYR5 ATPYR7
RUE1	Lower Austria; Rührsdorf	Williams/rootstock unknown	Standard tree, pyramidal shape, stem height 120 cm	30	Extensive	ATPYR5
RUE2	Lower Austria; Rührsdorf	Williams/rootstock unknown	Hedge, stem height 50–60 cm	10	Extensive	ATPYR7
RUE3	Lower Austria; Rührsdorf	Williams/rootstock unknown	Standard tree, pyramidal shape, stem height 120 cm	4	Extensive	ATPYR9
HH	Lower Austria; Haschhof, Klosterneuburg	Perry pear varieties/ Pyrodwarf, quince A, Kirchensaller	Standard tree, pyramidal shape, stem height 120 cm	5–20	Organic	ATPYR6 ATPYR9 ATPYR11 ATPYR12 ATPYR13 ATPYR14 ATPYR15
WB	Lower Austria; Wilhelmsburg	Williams/ Pyrodwarf	Spindle, stem height 40–50 cm	6	Integrated	ATPYR2 ATPYR9
		Große Landbirne/BA29	Spindle, stem height 40–50 cm	6		
		Schweizer Wasserbirne/BA29	Spindle, stem height 40–50 cm	6		
		Speckbirne/pear seedling	Standard tree pyramidal shape, stem height 180 cm	24		
RIE	Upper Austria; Ried i.d. Riedmark	Pichlbirne, Schweizer Wasserbirne, Grüne Wilawitzl/rootstocks unknown	Standard tree pyramidal shape, stem height 180 cm	Different tree ages	Extensive	ATPYR6 ATPYR9
GRO	Styria, Großwilfersdorf	Abbe Fetel/-rootstock not assessed	Spindle, stem height 40–50 cm	5	Integrated	ATPYR8

^*^Phytoplasma genotypes as described in Table 5

### Population dynamics of pear psyllids

In RUE1, RUE2 and THA (Table1), insect population dynamics were analysed from February 2013 until October 2014, at site HH from March 2012 until November 2013. Insects were caught every 1–3 weeks by beating tray method, using a white plastic tray (30 × 40 cm) to capture individuals. Psyllids were immediately sampled from the tray with a mouth aspirator. Per tree ten hits were performed (2 branches, 5 hits per branch), and in total, 10 trees per location were sampled at each time point. Insect species and seasonal stage of the pear psyllids (winterform, summerform) were determined by aid of a stereomicroscope according to Ossiannilsson (1992) and Burckhardt and Jarausch (2007). Representative numbers of the collected psyllids were individually analysed by PCR for phytoplasma presence. Numbers of PCR samples per insect species, season and location are illustrated in Tables 2 and 3.

**Table 2:** Nested PCR analysis of *C. pyri* and *C. pyricola* presented according to sampling (calendar) weeks and presumed insect stage (SF1, 2, 3, summerform individuals presumably 1st, 2nd, 3rd generation; WF-winterform specimens): N° of PCR-positive individuals/total N° of analysed individuals (percentage of PCR-positive individuals). 2013, 2014: Pooled data from all locations separated according to calendar years

*C. pyricola*	Site	SF 1–2 week 15–22	SF 1–2 week 23–28	SF 3 week 29–36	WF week 40–48	WF week 7–19	Total
	HH	1/48 (2.1%)	5/91 (5.5%)	8/132 (6.1%)	2/114 (1.8%)	4/44 (9.1%)	20/429 (4.6%)
	RUE1			0/2	0/1	1/7 (14.3%)	1/10 (10%)
	RUE2	0/39	0/24	2/8 (25%)	0/6	3/48 (6.3%)	5/125 (4%)
	**All sites**	**1/87 (1.1%),** 2013: 1/87 (1.1%) 2014:-	**5/115 (4.3%)** 2013: 5/115 (4.3%) 2014:-	**10/142 (7.0%)** 2013: 10/142 (7%) 2014:-	**2/121 (1.7%)** 2013: 1/50 (2%) 2014: 1/72 (1.4%)	**8/99 (8.1%)** 2013: 6/83 (7.2%) 2014: 2/16 (12.5%)	**26/564 (4.6%)**
***C. pyri***	Site	SF1 week 18–22	SF 2 week 23–28	SF 3 week 29–37	WF week 38–48	WF week 7–17	Total
	HH	0/12	0/10	1/5 (20%)			1/27 (3.7%)
	RUE1	0/75	0/34	9/96 (9.4%)	0/10	5/37 (13.5%)	14/252 (5.5%)
	RUE2	0/3	1/3 (33%)	0/2	0/1	0/3	1/12 (8.3%)
	THA	1/62 (1.6%)	0/27	4/42 (9.5%)	4/109 (3.7%)	15/205 (7.3%)	24/445 (5.4%)
	**All sites**	**1/152 (0.6%)** 2013: 1/100 (1%) 2014: 0/52	**1/74 (1.4%)** 2013: 1/35 (2.9%) 2014: 0/38	**14/145 (9.7%)** 2013: 11/86 (12.7%) 2014: 3/59 (5.1%)	**4/120 (3.3%)** 2013: 3/72 (3.8%) 2014: 1/48 (2.1%)	**20/245 (8.2%)** 2013: 4/35 (11.4%) 2014: 16/210 (7.6%)	**40/736 (5.4%)**

The average numbers for all sites and corresponding percentages are shown in bold

**Table 3:** Nested PCR analysis of *C. pyrisuga* according to insect stage (remigrants, emigrants): N° of PCR-positive individuals/total N° of analysed individuals (percentage of PCR-positive individuals)

Site	Remigrants	Emigrants	Total
HH	9/55 (16.4%)	–	9/55 (16.4%)
RUE1	5/39 (12.8%)	0/1	5/40 (12.5%)
RUE2	3/55 (5.5%)	–	3/55 (5.5%)
THA	3/60 (5%)	0/1	3/61 (4.9%)
**All sites**	**20/209 (9.6%)** 2013: 6/70 (8.6%) 2014: 14/109 (12.8%)	**0/2** 2013: 0/2	**20/211 (9.5%)**

The average numbers for all sites and corresponding percentages are shown in bold

### Cage transmission trials

All insects included in the transmission experiments were adults, field collected from March 2013 until March 2015 at RUE1, RUE2, HH and THA (Table 1) as described above. For species determination of living insects, each single individual was caged in a transparent petri dish and identified by aid of a stereo microscope using reference specimens and morphological characteristics according to Ossiannilsson (1992) and Burckhardt and Jarausch (2007). Foliated pear seedlings grown from seeds in our laboratory (cv. 'Williams' and cv. 'Bosc’s Flaschenbirne') cultivated at 21 °C served as test plants. Single plants were entirely covered with transparent cylindrical cages (diameter 9 cm, height 25 cm). On each test plant, 10 pear psyllid individuals were caged and allowed to feed for one week. After the trials, the test plants were cultivated under insect proof conditions in a cold greenhouse. Twice a year (until summer 2019), root samples were collected for PCR analysis. Numbers of transmission experiments for each pear psyllid species, insect sampling location and test season are illustrated in Table 4.

**Table 4:** Results of transmission experiments grouped according to calendar weeks and insect stage (SF-summerform; WF-winterform): N° of infected test plants/total N° of tests (percentage of infected plants)

***C. pyricola***	Site	SF week 18–22	SF week 23–28	WF week 40–48	WF week 7–17	Total
	HH	0/4	–	1/22 (4.5%)	0/16	1/42 (2.4%)
	RUE2	0/4	–	-	1/3 (33.3%)	1/7 (14.3%)
	**All sites**	**0/8**	–	**1/22 (4.5%)**	**1/19 (5.3%)**	**2/49 (4.1%)**
***C. pyri***		SF week 18–22	SF week 23–28	WF week 40–48	WF week 7–17	Total
	RUE1	0/4	0/11	–	–	0/15
	THA	0/1	0/2	3/15 (20%)	2/11 (18.0%)	5/29
	**All sites**	**0/5**	**0/13**	**3/15 (20%)**	**2/11 (18.1%)**	**5/44 (11.4%)**
***C. pyrisuga***		Emigrants		Remigrants		
	HH	–		1/9 (11.1%)		
	RUE2	–		0/1		
	THA	–		1/11 9.1%)		
	**Total**			**2/21 (9.5%)**		

### Phytoplasma detection in plants and insects

Plant samples from orchards consisted of pooled root material collected from 3 different sides of individuals trees (diameter 3–8 mm) in September and October 2012–2019. In case of pear seedlings from transmission experiments, 2–3 root pieces (diameter 2–5 mm) per plant were excised taking care of maintaining a functioning root system in order to keep the plants alive for further analyses. Plants from transmission experiments were tested twice a year, in spring and in autumn from 2014 to 2019. Roots were exhaustively washed by aid of a rough sponge. Each sample consisted of 1–2 cross sections of each root piece. DNA extraction from plant and insect samples was carried out by a CTAB—procedure (Maixner et al. 1995). The presence of phytoplasmas was detected by nested PCR with primers R16F2/R2 (Lee et al. 1993) and R16(X) f1/r1 (Lee et al. 1995). A reaction mixture of 20 µl contained 1 µl template preparation, 0.5 µM of each primer, 200 µM of each dNTP, 0.5U TopTaq DNA polymerase and 1 × PCR buffer (Qiagen, Erlangen, Germany). PCR was performed in an Eppendorf Mastercycler (Hamburg, Germany) performing 40 cycles with 45 s denaturation at 94 °C, 45 s annealing at 50 °C and 60 s extension at 72 °C. 1 µl PCR product diluted 1:100 served as template for the second run with identical parameters but using 1 × coral load PCR buffer and 54 °C annealing temperature. Positive samples were digested with *RsaI* (Promega, Madison, WI, USA) at 37 °C for 2.5 h (Seemüller and Schneider 2004) or directly sequenced by Sanger sequencing to differentiate between 16SrX phytoplasmas and to exclude unspecific products. PCR and RFLP products were stained with MidoriGreen (Nippon Genetics Europe, Dueren, Germany), separated on a 2% agarose gel and visualised under UV light. Plant samples originating from transmission experiments were in addition (in order to confirm results of nested PCR) analysed by qualitative qPCR (Christensen et al. 2004) using the SensiFAST Probe No Rox Kit (Bioline, London, UK) according to the producer’s instructions and a MIC thermocycler (Biomolecular Systems, Upper Coomera, Australia). 1 µl of template preparation was included in 20 µl reaction volumes.

### Strain characterisation

Phytoplasma-infected plant and insect samples were used for strain characterisation using the genes *aceF* and *imp.* Nested PCR for *aceF* was performed with primers AceFf1/r1 and AceFf2/r2 (Danet et al. 2011) or for improved PCR amplification with the modified '*Ca.* P. pyri' primers 5`-TAAAATTTGCTGATATTGGCG-3`(AceFpyri_f1), 5`-CATCTTTAATTTCATTAAAACTAG-3`(AceFpyri_r1), 5`-AGGTATTGATGAAGGAACTG-3`(AceFpyri_f2) and 5`-TAATTGCCTTCATAATAAAAG-3`(AceFpyri_r2). *Imp* was amplified using the primers IMPF2bis/R1bis and IMPF3pyr/r4pyrA (Danet et al. 2011) specific for '*Ca.* P. pyri'. PCR was performed as described above using 40 cycles with 60 s denaturation at 94 °C, 60 s annealing at 54 °C and 45 s extension at 72 °C and final elongation at 72 °C for 10 min. PCR fragments were sequenced by Sanger sequencing with sequencing primers AceFf2 and IMPF3pyr for *aceF* and *imp* gene, respectively. Multiple alignments were carried out with the obtained data and reference entries from the NCBI database using ClustalW in MEGA 7.0 (Kumar et al. 2016) followed by construction of phylogenetic trees. The evolutionary history was inferred using the neighbour-joining method, and bootstrapping using 1000 replicates was performed. For reasons of comparability, the designations of *aceF* and *imp* genotypes from previous studies were maintained and include *aceF* affiliations A10, A11, A12, A18-A20, A24 (Danet et al. 2011), A25 (Pavšič et al. 2014) and *imp* affiliations I14-I20, I27, I28 (Danet et al. 2011) and B3 (designated as I35; Bohunicka et al. 2018).

## Results

### Population dynamics and infection rates of pear psyllids

Analyses in pear orchards in Austria showed the presence of *C. pyri*, *C. pyricola* and *C. pyrisuga* in all 4 orchards (data for 2013: Fig. 1, data for 2014: Online resource 1, data for 2012 HH: Online resource 2). Abundance of *C. pyri* and *C. pyricola* varied greatly between the test orchards; in THA and RUE1 almost exclusively *C. pyri* individuals were captured, in HH and RUE2 almost exclusively *C. pyricola*. The overwintering generation of *C. pyri* (winterform individuals) occurred between calendar weeks 38 and 17 and the overwintering generation of *C. pyricola* between calendar weeks 40 and 16. Summerform individuals of *C. pyri* were observed between weeks 18 and 37, and insect captures showed 3 summer generations both in RUE1 and in THA. Summerform *C. pyricola* occurred between weeks 15 and 37, and the data also indicated the presence of 3 summerform generations in HH and RUE2, although first and second generation overlapped*. C. pyrisuga* remigrants were present in orchards between weeks 12 and 21, and springtime generation adults were rarely captured between weeks 21 and 24.

**Fig. 1:**
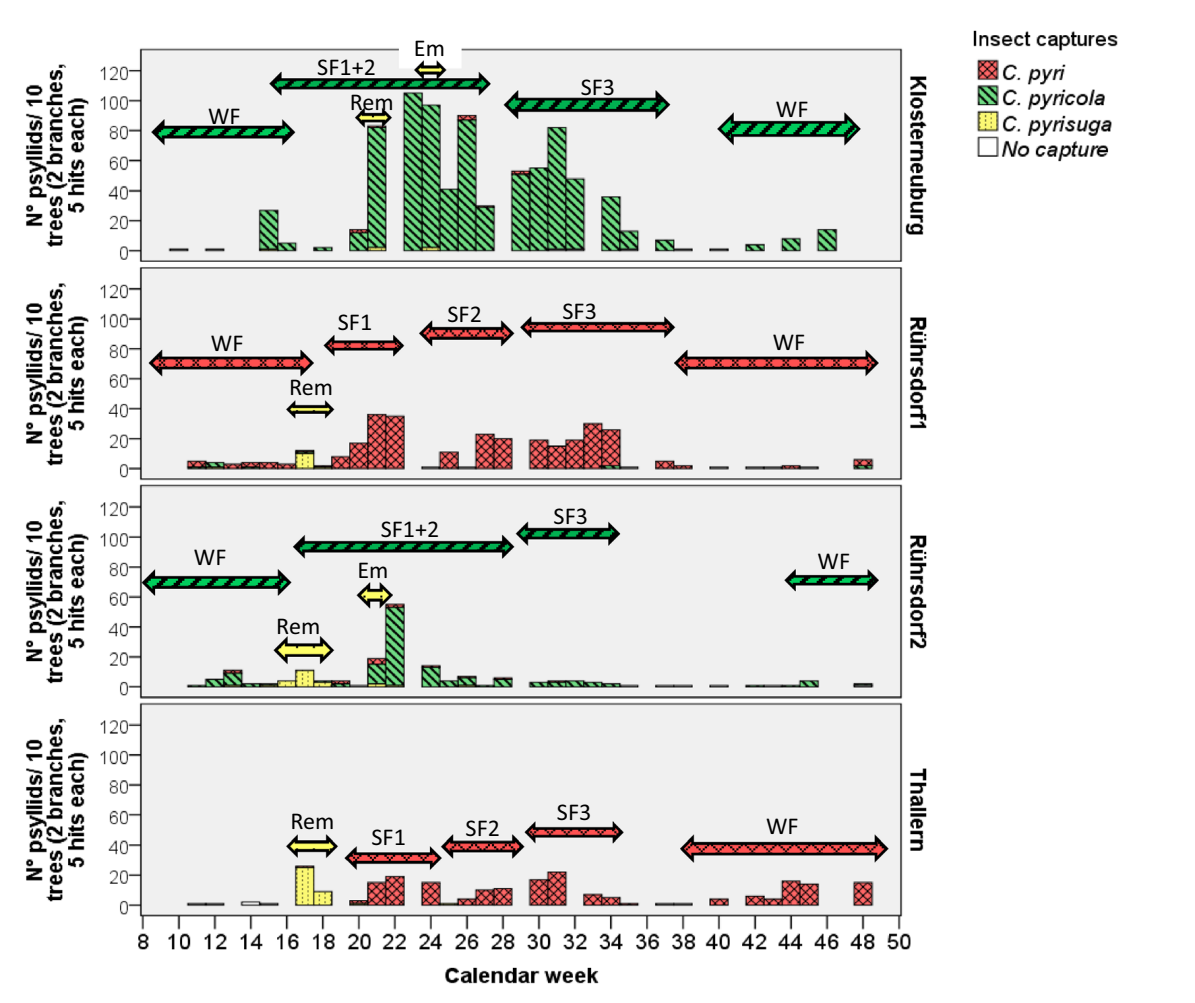
Captures of pear psyllids at each sampling location in the course of the year 2013. Numbers represent total captures of insects on 10 trees (2 branches with 5 hits each per tree). WF-winterform individuals, SF1,2,3, summerform individuals presumably 1st, 2nd, 3rd generation, Rem- *C. pyrisuga* remigrants, Em-*C. pyrisuga* emigrants

Rates of PCR-positive pear psyllids are illustrated in Tables 2 and 3. Data for each insect species were summarised according to the calendar weeks in which the insects were recorded (presumptive insect generations as illustrated for 2013 in Fig. 1). Overall infection rates for *C. pyri* were 5.4%, for *C. pyricola,* 4.6%, for *C. pyrisuga* remigrants 9.6% and for *C. pyrisuga* emigrants 0%. For *C. pyri* and *C. pyricola,* the rates of PCR-positive individuals in all orchards varied greatly in the course of the year. The highest rates of infected *C. pyricola* were observed between late summer and autumn (weeks 29–36, overall infection rate 7.0%) and in late winter (week 7–17, overall infection rate 8.1%). For *C. pyri,* we observed the highest infection rates from late summer to autumn (weeks 29–37, overall infection rate 9.7%) and in late winter to early spring (overall infection rate 8.2%).

### Cage transmission trials

As illustrated in Table 4, the cage transmission experiments resulted in in phytoplasma transmissions by all three pear psyllid species. Data were grouped in accordance with calendar weeks and putative insect generations. Experiments with the winterform individuals of *C. pyricola* lead to transmission rates of 4.5% and 5.3% for weeks 40–48 and 7–17, respectively. For winterform individuals of *C. pyri,* transmission rates of 20 and 18.1% were recorded for weeks 40–48 and 7–17. None of the experiments including summerform individuals of *C. pyri* and *C. pyricola* carried out between calendar weeks 18 and 28 resulted in phytoplasma transmission*. C. pyrisuga* remigrants transmitted the phytoplasma in 2 out of 21 experiments (9.5%).

### Characterisation of phytoplasma types

**Fig. 2:**
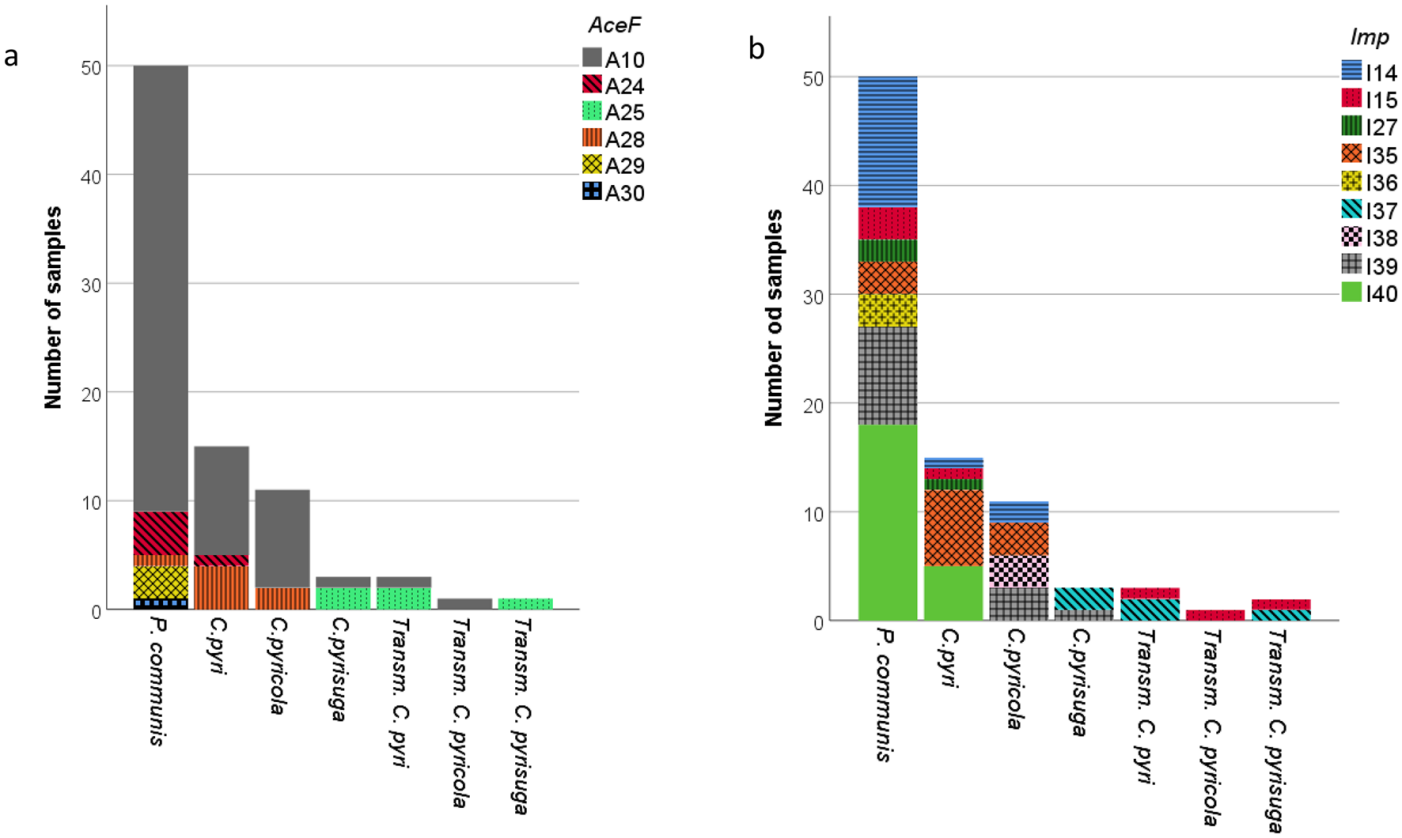
Occurrence of *imp* (**a**) and *aceF* (**b**) haplotypes of ‘*Ca*. P. pyri’ in *P. communis*, the three vector species (*C. pyri*, *C. pyricola* and *C. pyrisuga*) and host plants from transmission experiments (Transm.) with the three vectors species

MLST of in total 85 samples, based on the analysis of the genes *aceF* and *imp,* revealed 6 different genotypes in *aceF* and 9 different genotypes in *imp* genomic loci (Fig. 2). The comparison between Austrian isolates and NCBI database entries revealed 3, so far undescribed, *aceF* genotypes (in THA-38-pyrus A28 named according to the nomenclature of Danet et al. (2011), in THA-39-pyrus A29 and in HH-5224-pyrus A30) and 4 so far undescribed *imp* genotypes (in THA-39-pyrus I36, in HH-284-pyrisuga I37, in HH-1751-pyricola I38 and in HH-1766-pyrisuga I39). All other *aceF* and *imp* sequences have been published previously. Genotypes were named according to strains described by Bohunicka et al. (2018), Danet et al. (2011) and Pavšič et al. (2014) as shown in Fig. 2 and Table 5. The combination of *aceF* and *imp* gene analysis allowed the discrimination between 15 different phytoplasma types (Table 1, Table 5, Fig. 2 and Online resource 3). Out of the 15 types, 13 were present in pear trees. Eight types were ascertained in *C. pyri* (samples originating from HH and RUE1 + 2 and THA), six in *C. pyricola* (collected in HH and RUE1 + 2) and 2 in *C. pyrisuga* (from HH). In case of the transmission experiments, two types were identified in experiments with *C. pyri* (from THA), one in each case in experiments with *C. pyricola* and *C. pyrisuga* (both from HH). In one transmission experiment with *C. pyrisuga* (from THA), however, the successful amplification of only one gene (*imp*) was obtained. In this case, an unambiguous ascription of the phytoplasma to a type was only partially possible (Online resource 3).

**Table 5:** Classification of phytoplasma genotypes based on *aceF* and *imp* sequences of ‘*Ca.* P. pyri‘

Genotype	*aceF* genotype^1^	*aceF* accession	*aceF* identical accession^2^	*imp* genotype^1^	*Imp* accession	*imp* identical accession^2^	Strain	Hosts^3^	Region^4^
ATPYR1	A28^5^	MW456643	–	I35^5^ (B3, covers 89%)	MW456649	MF374928	THA-38-pyrus	Pear *C. pyri* *C. pyricola*	RUE1 RUE2 THA
ATPYR2	A29^5^	MW456644	–	I36^5^ (G, covers 70%)	MW456650	–	THA-39-pyrus	Pear	THA WB
ATPYR3	A25 (D1040-07)	MW456645	KF849468	I37^5^ (A1, covers 89%)	MW456651	–	HH-284-pyrisuga	*C. pyrisuga* *C. pyri* Pear	HH THA
ATPYR4	A10 (PD)	MW456646	FN598177	I38^5^	MW456652	–	HH-1751-pyricola	*C. pyricola*	HH
ATPYR5	A10 (PD)	MW456646	FN598177	I35^5^ (B3, covers 89%)	MW456649	MF374928	HH-1759-pyricola	Pear *C. pyri* *C. pyricola*	HH RUE1 THA
ATPYR6	A10 (PD)	MW456646	FN598177	I39^5^	MW456653	–	HH-1766-pyricola	Pear *C. pyrisuga* *C. pyricola*	HH RIE
ATPYR7	A10 (PD)	MW456646	FN598177	I40^6^ (Mass1)	MW456654	HG737344	THA-14-pyrus	Pear *C. pyri*	RUE1 RUE2 THA
ATPYR8	A10 (PD)	MW456646	FN598177	I27 (PIHRKT1)	MW456655	FN600728	RUE1- 180-pyri	Pear *C. pyri*	RUE1, GRO
ATPYR9	A10 (PD)	MW456646	FN598177	I14 (PD280-4)	MW456656	FN600721	RUE3-1-pyrus	Pear *C. pyri* *C. pyricola*	HH RIE RUE1 RUE2 RUE3 WB
ATPYR10	A24 (TR1)	MW456647	FN598183	I35^5^ (B3, covers 89%)	MW456649	MF374928	RUE1-820-pyri	*C. pyri*	RUE1
ATPYR11	A10 (PD)	MW456646	FN598177	I15 (PD-20)	MW456657	FN600722	HH-1771-pyri	Pear *C. pyri* *C. pyricola*	HH THA
ATPYR12	A30	MW456648	–	I39^5^	MW456653	–	HH-5224-pyrus	Pear	HH
ATPYR13	A24 (TR1)	MW456647	FN598183	I39^5^	MW456653	–	HH-5244-pyrus	Pear	HH
ATPYR14	A24 (TR1)	MW456647	FN598183	I14 (PD280-4)	MW456656	FN600721	HH-5253-pyrus	Pear	HH
ATPYR15	A24 (TR1)	MW456647	FN598183	I15 (PD-20)	MW456657	FN600722	HH-5235-pyrus	Pear	HH

^1^*aceF* and *imp* genotypes based on previously published nomenclature (Danet et al. 2011; Dermastia et al. 2018). Genotypes published by Bohunicka et al. (2018) and partly covering the *imp* genes identified here and previously published strains are indicated in brackets

^2^Accession numbers and corresponding strains with 100% sequence identity

^3^Representative hosts are indicated

^4^Regions: Lower Austria: Haschhof, Klosterneuburg (HH), Thallern (THA), Rührsdorf 1–3 (RUE1-3), Wilhelmsburg (WB); Upper Austria: Ried in der Riedmark (RIE); Styria: Großwilfersdorf (GRO)

^5^No corresponding entry in the NCBI database

^6^Suggested *Imp* number for previously published genotypes

Neighbour-joining calculated phylogenetic distances among *aceF* sequences of the Austrian strains found in this study, and reference strains from the NCBI database are visualised in Online resource 4. Based on the compared *aceF* genomic region (Online resource 4), the *aceF* sequences identical to TR1 (A24; D119-08); PD (A10; D877-07); and D1040-07 (A25) could be identified also in this study, while the *aceF* sequence of the strain THA-38-pyrus (A28) described here is > 99.8% identical to the *aceF* published sequence of strain TR1 (A24), separated by a single SNP. The *aceF* sequence of HH-5224-pyrus (A30) is 99.7% identical to PD (A10) corresponding to two SNP. The Austrian strain THA-39-pyrus (A29) is further separated from other strains with 4 SNP differentiation to the closest relatives TR1 (A24), corresponding to 99.4% identity. *AceF* sequence of strains PD and D877-07 (A10) accounted for 79.1% of the analysed isolates. The so far uncharacterised *aceF* sequences A28, A29 and A30 represented 8.2%, 3.5% and 1.2% of the isolates, respectively. In addition, in at least five pears in HH in 2019 double infections with *aceF* A10 and A24, which are not included in Fig. 2 and Online resource 4, could be observed.

Genotyping applied on the *imp* locus revealed higher variability than the *aceF* region. Out of the five so far uncharacterised *imp* sequences (Table 5, Fig. 2b), *Imp* I35 of strain THA-38-pyrus shares identical sequence with the recently described Czech strain AB860 (B3), which covers THA-38-pyrus to 89% and is closely related to strains Mass1 (designed as I40) and AA895 (B1), PD33lib (I18) and PD (I19) separated by 2 each, 4 and 10 SNPs, respectively. The *imp* sequence of strain THA-39-pyrus (I36) clusters together with the recently described Czech strain AA304 (G), with that it shares identical sequence. However, strain AA304 covers only 70% of the *imp* sequence of strain THA-39 published here and THA39 is separated from strain PIHRZG1 (I28) by 23 SNPs. *Imp* I37 of strain HH-284-pyrisuga is identical to AA284 (A1), which covers 89% of the HH-284-pyrisuga *imp* I37 sequence. *Imp* I37 is related to strain (A2) (1 SNP distance) and separated by 16 and 17 SNPs from strains PD36AZ (I16) and P1HRKT1 (I27), respectively. The *imp* of strain HH-1751-pyricola (I38) shares a common ancestor with THA-38-pyrus (I35) and Mass1 (I40), PD33lib (I18) and PD (I19) separated by at least 12 SNPs. The *imp* of strain HH-1766-pyricola (I39) is related to strains PD-20 (I15) and PD280-4 (I14), separated by 5 and 6 SNPs, respectively. The *imp* of genotype Mass1 (assigned *imp* name I40) was predominant in Austria and accounted for 27.1% of analysed isolates. The five *imp* genotypes found in this study THA-38-pyrus (I35), THA-39-pyrus (I36), HH-284-pyrisuga (I37), HH-1751-pyricola (I38) and HH-1766-pyricola (I39) represented 15.3%, 3.5%, 5.9%, 3.5% and 15.3% of all isolates, respectively. In addition, in at least three pears in HH in 2019 double infections with *imp* I14 and I39, which are not included in Fig. 2 and Online resource 4, could be observed.

## Discussion

Investigations in pear orchards allowed the conclusion that *C. pyri*, *C. pyricola* and *C. pyrisuga* are regularly present in pear orchards in Lower Austria. Significant populations of the polyvoltine psyllids *C. pyri* and *C. pyricola* were detected during the entire monitoring period. The highest insect densities were present in all orchards and sampling years approximately between May and August (calendar weeks 18 to 33), but relevant numbers of winterform individuals in late autumn and early spring were also recorded. Interestingly, we observed remarkable differences among the orchards with regard to presence of *C. pyri* and *C. pyricola*. The two species never co-occurred to the same extent; in Thallern and Rührsdorf 2, nearly solely *C. pyri* was captured, and in Klosterneuburg and Rührsdorf 1, *C. pyricola* was clearly predominant. The reason for this imbalance is not clear.

Remigrant *C. pyrisuga* adults were observed on *P. communis* in all orchards in all test years from middle of March onwards. Captures of new generation adults were much rarer, and only single individuals of the new generation were caught during the monitoring period (Fig. 1, Online resources 1 and 2). Substantially lower catches of springtime generation insects as compared to remigrants were also recorded in a previous investigation with the migratory psyllid species *C. pruni* (Maier et al. 2013). Possibly, the new generation psyllids leave the orchards relatively soon after hatching,

PCR analysis of *C. pyri* and *C. pyricola* individuals showed that phytoplasma-infected specimens are present in orchards all year round (Table 2). Infection rates, however, varied greatly between periods of the year and assumed insect generations. Whereas in spring and in early summer insects, if at all, were only sporadically infected, individuals captured between calendar weeks 29–36 (mid of July until mid of September), presumably ascribable to the 3^rd^ summer generation, showed significant infection rates in all test orchards (except in Rührsdorf 2 with few tested individuals). Individuals belonging to the overwintering generation captured between weeks 40–48 (beginning of October until end of November) were infected at a lower rate as compared to the 3^rd^ summer generation, but percentages of infected individuals rose again to levels to the same extent as the 3rd summer generation in the course of winter (Table 2). A very similar fluctuation of infection rates was reported for *C. pyri* in Spain. Just as in our study, low infection rates were recorded between June and August followed by a significant increase in September and some decrease in October. The decrease in October was attributed to the emergence of the overwintering generation (Garcia Chapa et al. 2005; Sabaté et al. 2018). The observed fluctuation in the course of the year can probably be attributed to two causes. First, a seasonal variation of phytoplasma presence in the above-ground parts of the trees has been shown in previous studies. In wood and leaf samples collected from infected trees in spring, the pathogen was not detected or detected at a low rate only. It is presumed that due to the inactivation of the sieve tubes during winter, the phytoplasmas mainly survive in the roots of the plants. In spring, the pathogen recolonises the aerial parts of the tree, but colonisation of the newly grown leaves takes some time (Seemüller et al. 1984b; Errea et al. 2002). It may be assumed that psyllids feeding on weakly infected parts of the plant in spring are infected to a substantially lesser degree than individuals feeding on trees with high phytoplasma titres in late summer or autumn. Moreover, a study analysing the accumulation of ‘*Ca.* P. prunorum’ in *C. pruni* showed a constant phytoplasma multiplication in the insect bodies until a maximum phytoplasma load was reached approximately 85 days after acquisition (Thébaud et al. 2009). In our study, the overwintering generation of *C. pyri* and of *C. pyricola* was present for 5–6 months, whereas the summer generations stayed at maximum for 9 weeks. Thus, especially for the overwintering generation a relevant enrichment of the phytoplasmas in the insect bodies can be presumed which is likely to be the reason for the observed increase of PCR-positive *C. pyri* and *C. pyricola* winterform individuals from late autumn until early spring.

As illustrated in Table 4, the transmission experiments carried out in the current study led to successful pathogen transmission by *C. pyri, C. pyricola* and *C. pyrisuga*. To our knowledge, this is the first experimental confirmation of the vectoring ability of *C. pyrisuga* and also the first report of simultaneous pathogen transmission by *C. pyri, C. pyricola* and *C. pyrisuga* in one region. So far, successful transmission experiments have only been reported for *C. pyri* from several countries of continental Europe, and *C. pyricola* was confirmed as a vector in England and in North America (Jarausch et al. 2019a).

Transmission experiments with winterform individuals led to transmission rates of up to 20% for *C. pyr*i, and around 5% for *C. pyricola.* In contrast, no successful phytoplasma transmission by summerform, *C. pyri* and *C. pyricola,* captured between May and July (weeks 18 and 28), was recorded. Lower infectivity of spring and early summer generations has already been reported previously for *C. pyri* (Carraro et al. 2001). The enhanced infectivity of winterform individuals can on the one hand be explained by the already mentioned higher rates of PCR-positive individuals. Additionally, however, the longer lifespan of the winterform psyllids likely allows the accumulation of higher phytoplasma loads in the insects and their salivary glands resulting in increased transmission rates. Unfortunately, transmission experiments with individuals collected in late summer and autumn (3rd summer generation) are lacking in the current study. Based on insect infection rates, however, significant transmission rates seem to be likely as previously reported by Carraro et al. (2001).

The test plants in our study were cultivated under laboratory conditions, and their growth state might therefore differ from plants in natural conditions. Thus, from our study it remains unclear whether and to which extent the state of the pear plants under field conditions in late autumn and winter influences the phytoplasma establishment. Carraro et al. (2001) observed no transmission during dormancy but did, however, observe transmission from budbreak onwards. In our recent study on ‘*Ca* P. prunorum’ and *C. pruni,* successful phytoplasma transmission to apricot and plum trees in BBCH stages 03–09 was recorded (Riedle-Bauer et al. 2019). It is thus likely that in the field, the pathogen spread takes place from the start of tree development in spring onwards. Further experiments with trees cultivated outdoors, however, are required to determine to which extent phytoplasma transmission occurs during dormancy and to which extent the physiological status of the trees can influence the transmission.

The actual results underline the role of vector transmission for pathogen spread and simultaneously demonstrate the difficulties in view of effective vector control and disease management. The presence of several vector species with different biological and vectoring characteristics in one orchard results in a high risk of phytoplasma transmission all year round (except maybe in late spring and early summer). According to our experiments and literature data (Carraro et al. 2001), a high risk of phytoplasma transmission via *C. pyri* and *C. pyricola* is in late summer to autumn and particularly in late winter and early spring. Between harvest and swelling of inflorescence buds, however, the control of pear psyllids by insecticides is not homologised in many countries, for instance, in Austria (Bundesamt für Ernährungssicherheit 2021). Thus, the reduction of phytoplasma spread by *C. pyri* and *C. pyricola* must rely on a perfect tuning of treatment schedules during the vegetation period, a careful selection of applied insecticides and alternative products as well as on the support and protection by beneficials. However, less stringent legal constraints would probably allow a more efficient PD management (Belien et al. 2013).

In addition to the limited possibilities for control of *C. pyri* and *C. pyricola,* our study provides evidence that also the univoltine migratory psyllid *C. pyrisuga* efficiently transmits the pathogen. Due to its low population densities, *C. pyrisuga* is not a pest on its own and therefore it is currently not included in pear psyllid control strategies. Its role as phytoplasma vector might have been underestimated so far. In any case, apart from the status concerning homologised plant protection products, control of remigrating psyllid vectors, and thus phytoplasma spread by them, insecticide applications in early spring is difficult to achieve. It is hampered by cool temperatures, early developmental stages of the trees and the fact that remigration lasts for several weeks. Furthermore, the first tree(s) in an orchard reached by an infectious remigrant can only be protected by insecticides that not only reduce vector populations, but also directly influence pathogen transmission. For the latter, the treatment should act in less time than the inoculation access period, which is likely to be only the case for a few insecticidal compounds (e.g. with a knock down effect) or possibly for repellents (Paleskić et al. 2017).

All in all successful PD management by insecticides seems to be hardly feasible due to vector presence all year round, especially in times of a general trend towards reduced pesticide input. Naturally occurring predators can make a valuable contribution to psyllid control (Gajski et al. 2021) and their presence in orchards can be promoted, e.g. by a species rich flora in the surrounding of the orchards (Cross et al. 2010). Nevertheless, research on additional control strategies such as tolerant or resistant rootstocks (Seemüller et al. 1998; Jarausch et al. 2019b) is urgently needed.

One aim of the multilocus sequence analysis carried out in this study was to improve insight into phytoplasma epidemiology, more precisely into the role of transmission by propagation material versus spread by infectious vectors. Unfortunately, however, the obtained data are limited in this respect. We used here modified *aceF* primers for *Ca.* Phytoplasma pyri and thereby obtained more *aceF* sequences as with the originally published primers (Danet et al. 2011). But, all in all, we achieved a relatively low number of successful transmission experiments; for part of the samples, we were still unable to amplify *imp* and *aceF* marker genes, and insect sampling was restricted to four locations only. Nevertheless, the marker gene evaluation allowed the identification of pears in HH with clear double infections. The infection path could not be determined in this case.

The more common *aceF* and *imp* genotypes identified in the polyvoltine psyllids *C. pyri* and *C. pyricola,* A10, A28, I35, I39 and I40 are reflecting the types commonly found in pears. By contrast, genotypes such as I38 and I37 have solely been found in *C. pyricola* and *C. pyrisuga* and genotypes A29, A30, I27 and I36 have been found only in pears. In pears, also I15 is clearly overrepresented. I37 is strongly overrepresented n *C. pyrisuga,* and together with I15, in the transmission experiments with all three psyllid species. While this proves that this genotype can be principally transmitted to pears, it is unclear whether under- and overrepresentation of some genotypes in plants, insects and the transmission experiments is pure coincidence (especially in *C. pyrisuga*, where only three insects could be fully characterised), reflecting a distinct transmission probability, plant history or a high overall variability of PD, and a limited sampling set for the variation. The high overall variability is also supported by a recent publication (Bohunicka et al. 2018) from the neighbouring Czech Republic identifying 17, including several *imp* genotypes, which were undescribed before. Also, in our study both novel *aceF* and *imp* genotypes imply a still unexploited diversity of PD genotypes or a very high diversity.

## Supplementary Information

Below is the link to the electronic supplementary material.

Supplementary file1 (PDF 308 kb)

## Data Availability

All sequence data obtained in the study have been deposited at NCBI gene bank under the accession numbers MW456643—MW456657. Reference material is available from the authors upon request.
