# Supplementary material for: Vector transmission and epidemiology of ‘Candidatus Phytoplasma pyri’ in Austria and identification of Cacopsylla pyrisuga as new pathogen vector
Source: J Plant Dis Prot (2006). 2022 Feb 6;129(2):375–86. doi: 10.1007/s41348-021-00526-y (PMC8979874; doi:10.1007/s41348-021-00526-y)

Online resource 1:

Vector transmission and epidemiology of '*Candidatus* Phytoplasma pyri' in Austria

Journal of Plant Diseases and Protection

Monika Riedle-Bauer[1], Caroline Paleskić[1,2], Christina Schönhuber[2], Martina Staples[1], Günter Brader[2*]

[1]Federal College and Research Institute for Viticulture and Pomology Klosterneuburg, Wienerstraße 74, 3400 Klosterneuburg, Austria; Monika.Riedle-Bauer@weinobst.at

[2]Austrian Institute of Technology, Konrad-Lorenz-Straße 24, 3430 Tulln, Austria

[*] Corresponding author: guenter.brader@ait.ac.at; phone: +43(0) 50550-4669; fax: +43(0) 50550-3666

Captures of pear psyllids at the locations Rührsdorf 1, Rührsdorf 2 and Thallern, Lower Austria, in the course of the year 2014. Numbers represent total captures of insects on 10 trees (2 branches with 5 hits per tree).

WF-winterform individuals, SF1,2,3, Summerform individuals presumably 1st, 2nd, 3rd generation, Rem-remigrants, Em-Emigrants.

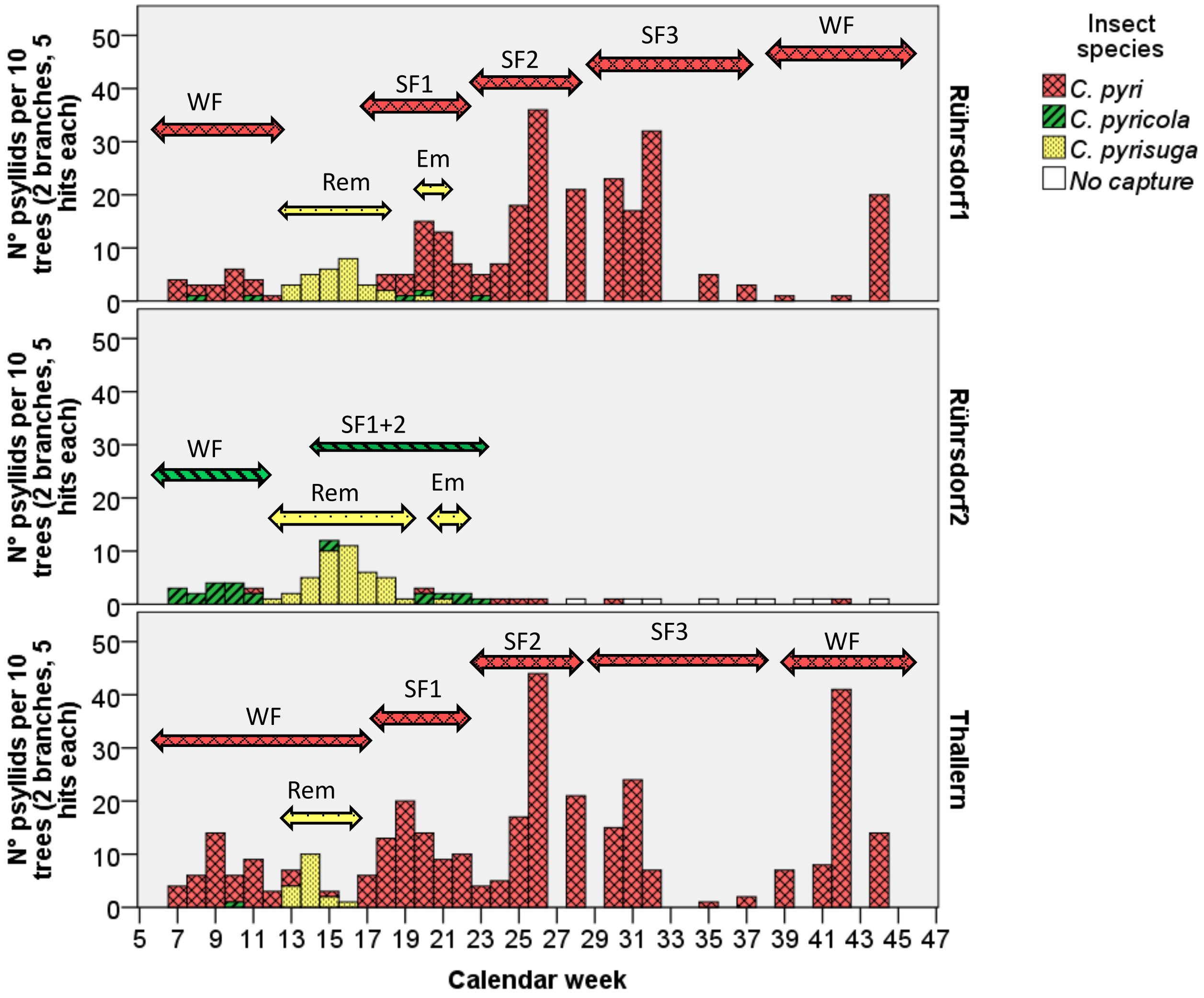

Online resource 2:

Vector transmission and epidemiology of '*Candidatus* Phytoplasma pyri' in Austria
Journal of Plant Diseases and Protection
Monika Riedle-Bauer[1], Caroline Paleskić[1,2], Christina Schönhuber[2], Martina Staples[1], Günter Brader[2*]
[1]Federal College and Research Institute for Viticulture and Pomology Klosterneuburg, Wienerstraße 74, 3400 Klosterneuburg, Austria; Monika.Riedle-Bauer@weinobst.at
[2]Austrian Institute of Technology, Konrad-Lorenz-Straße 24, 3430 Tulln, Austria
[*] Corresponding author: guenter.brader@ait.ac.at; phone: +43(0) 50550-4669; fax: +43(0) 50550-3666

Captures of pear psyllids in Klosterneuburg in 2012. Numbers represent total captures of insects on 10 trees (2 branches with 5 hits each per tree).

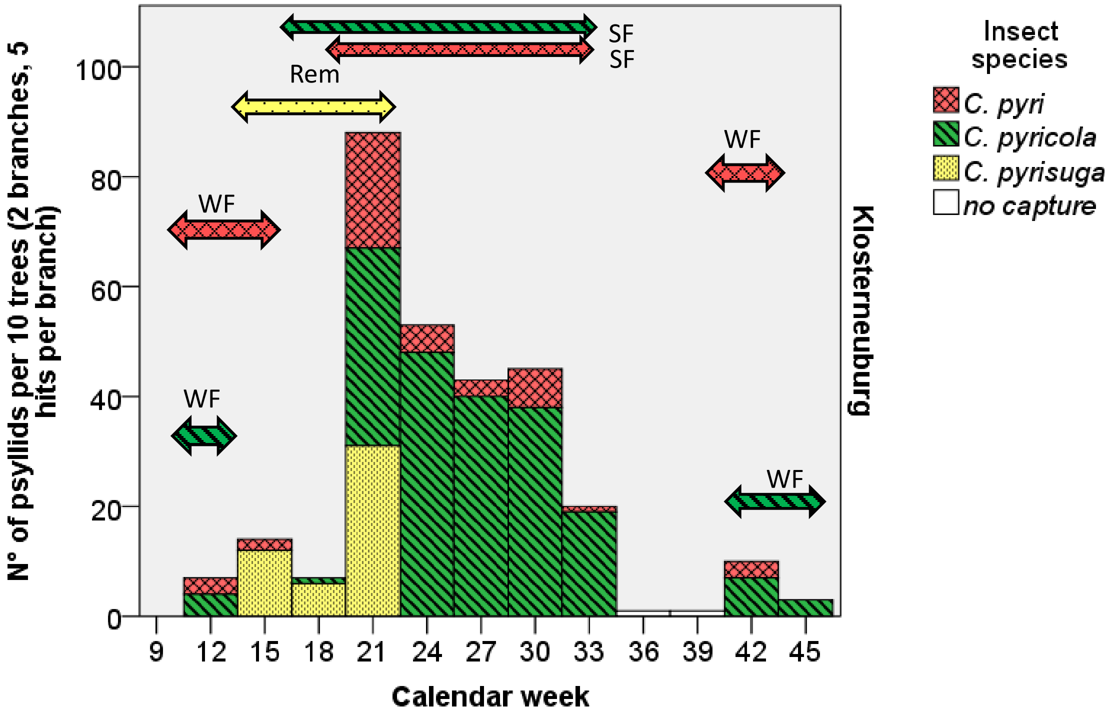

Online resource 3:

Vector transmission and epidemiology of ‘*Candidatus* Phytoplasma pyri’ in Austria

Journal of Plant Diseases and Protection

Monika Riedle-Bauer[1], Caroline Paleskić[1,2], Christina Schönhuber[2], Martina Staples[1], Günter Brader[2*]

[1]Federal College and Research Institute for Viticulture and Pomology Klosterneuburg, Wienerstraße 74, 3400 Klosterneuburg, Austria; Monika.Riedle-Bauer@weinobst.at

[2]Austrian Institute of Technology, Konrad-Lorenz-Straße 24, 3430 Tulln, Austria

[*] Corresponding author: guenter.brader@ait.ac.at; phone: +43(0) 50550-4669; fax: +43(0) 50550-3666

Characterisation of phytoplasma types by aid of the genes *aceF* and *imp:* Phytoplasma types detected in *P. communis*, the three vector species (*C.pyri, C.pyricola, C.pyrisuga*) and host plants from transmission experiments (Transm.) with the three vectors species.

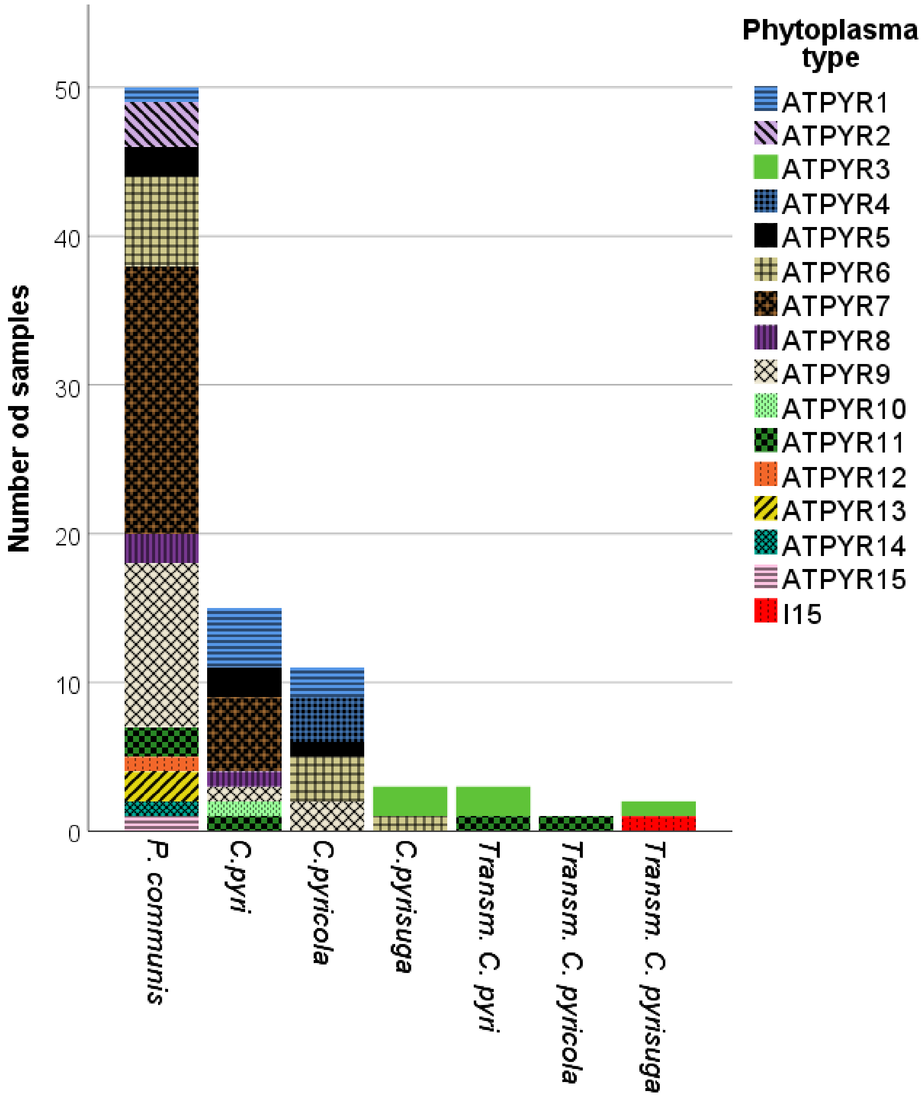

Online resource 4:

Vector transmission and epidemiology of ‘*Candidatus* Phytoplasma pyri’ in Austria

Journal of Plant Diseases and Protection

Monika Riedle-Bauer[1], Caroline Paleskić[1,2], Christina Schönhuber[2], Martina Staples[1], Günter Brader[2*]

[1]Federal College and Research Institute for Viticulture and Pomology Klosterneuburg, Wienerstraße 74, 3400 Klosterneuburg, Austria; Monika.Riedle-Bauer@weinobst.at

[2]Austrian Institute of Technology, Konrad-Lorenz-Straße 24, 3430 Tulln, Austria

[*] Corresponding author: guenter.brader@ait.ac.at; phone: +43(0) 50550-4669; fax: +43(0) 50550-3666

Phylogenetic relationship of identified *aceF* (a) and *imp* (b) strains compared to strains of NCBI database. The optimal tree with the sum of branch length = 0.0988 (a) and 0.3276 (b) are shown. The percentage of replicate trees in which the associated taxa clustered together in the bootstrap test (1000 replicates) are shown next to the branches. Strains identified in this study are marked with an asterisk. In brackets after the strain name the A (*aceF*) and *imp* (I) nomenclature of Danet et al. (2011) and, if applicable, the A-K nomenclature of Bohunicka et al. (2018) is indicated.

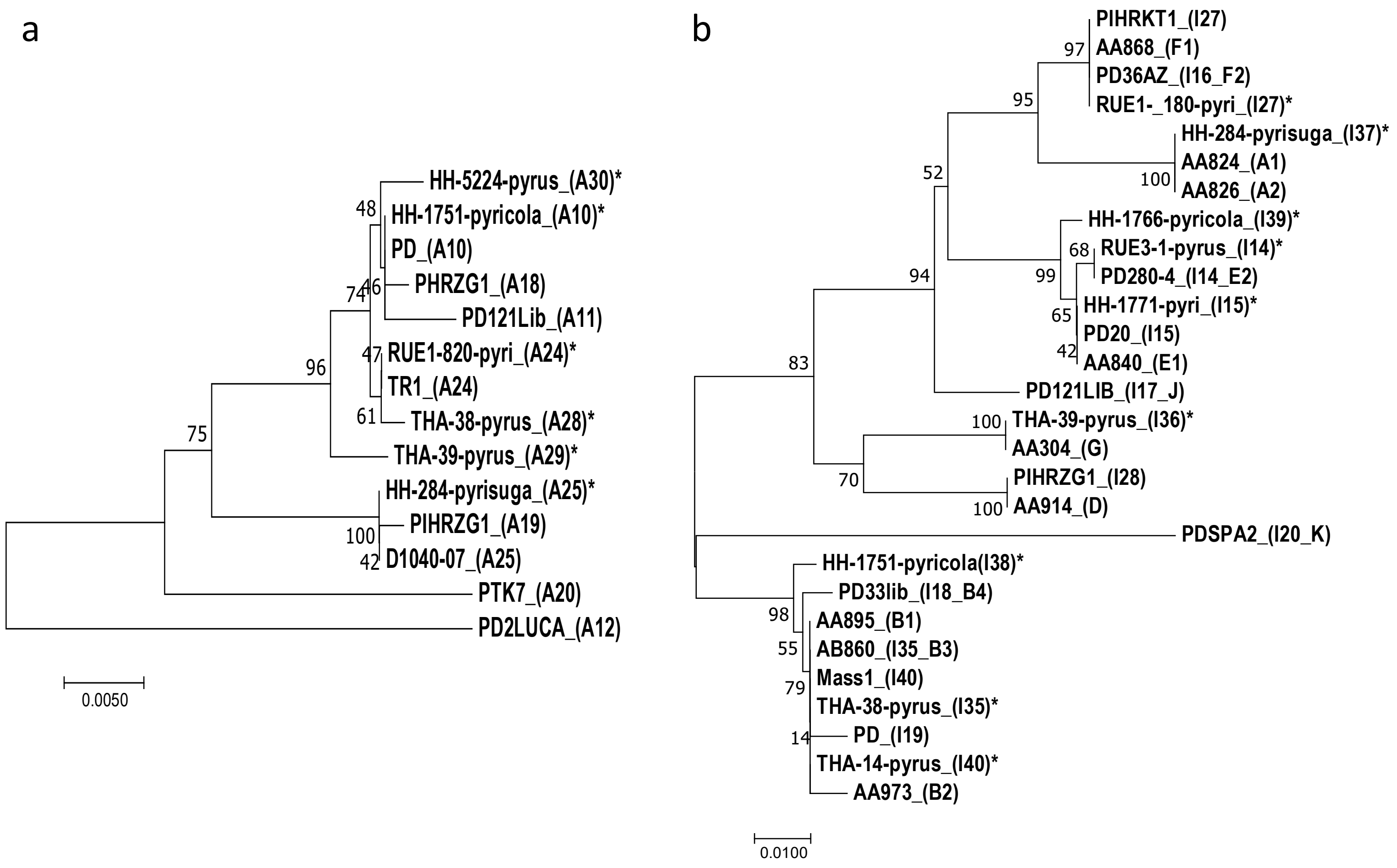

Supplement: Supplementary file 1 — Supplementary file1 (PDF 308 kb) [file 41348_2021_526_MOESM1_ESM.pdf]
